# Trauma, Emotional Neglect, and Developmental Vulnerability in Children: Evidence from Albania

**DOI:** 10.3390/bs15121608

**Published:** 2025-11-21

**Authors:** Anila Sulstarova, Blerta Bodinaku, Skerdi Zahaj, Gerda Sula, Greta Hysi

**Affiliations:** Department of Psychology, Faculty of Social Sciences, University of Tirana, 1001 Tirana, Albania; skerdi.zahaj@unitir.edu.al (S.Z.); gerda.sula@unitir.edu.al (G.S.); greta.hysi@unitir.edu.al (G.H.)

**Keywords:** human trafficking, attachment disruption, PTSD, caregiving breakdown, emotional safety, relational trauma, forced migration

## Abstract

Background: Children in Albania and the wider Balkan region are often exposed to subtle yet persistent forms of emotional absence, parentification, and silencing. These relational harms are culturally normalized and rarely identified as neglect, but they create significant developmental vulnerabilities and increase the risk of exploitation, including trafficking. Methods: This qualitative study involved 30 participants, including 16 frontline professionals (psychologists, social workers, and legal staff) and 14 survivors of trafficking. Data were collected through semi-structured, trauma-informed interviews and focus groups between December 2024 and March 2025. Reflexive thematic analysis was applied to identify emotional and relational patterns contributing to vulnerability, with attention to cultural contexts and gendered dynamics. Results: Three interrelated themes were identified: (1) emotional absence: children adapt to caregivers’ physical presence but emotional unavailability, leading to self-effacement and diminished entitlement to care; (2) parentification: children assume emotional caregiving roles, often regulating parents’ wellbeing; and (3) silencing: emotional expression becomes equated with shame or punishment, producing long-term relational invisibility. Conclusions: Early relational harms describe developmental conditions that may heighten susceptibility. Prevention and intervention should integrate attachment-based family assessments, early childhood screening, trauma-informed training for professionals, and culturally adapted approaches to break cycles of invisible harm and strengthen children’s emotional safety.

## 1. Introduction

The intersection of childhood adversity and human trafficking has received growing scholarly attention, particularly within the frameworks of trauma, disrupted attachment, and developmental risk (Bowlby, 1969). While multiple studies have emphasized the role of poverty, violence, and institutional neglect in producing vulnerability, less attention has been paid to the nuanced, often invisible forms of relational harm that precede exploitation, especially within family contexts that appear, on the surface, materially functional (Bifulco & Schimmenti, 2019).

This dimension becomes especially salient in regions where trafficking is not only an external threat but also a symptom of deeper intergenerational ruptures in caregiving and emotional recognition. In Albania and the wider Balkan region, a complex history of political upheaval, economic instability, and intergenerational trauma has shaped parenting styles, gender roles, and cultural expectations regarding emotional expression and dependence. Within these contexts, children may grow up in environments where emotional needs are minimized, discouraged, or punished despite the absence of overt abuse or neglect. Such conditions may remain unrecognized by social services, families, and the children themselves due to their normalization and cultural embedding.

Recent literature has begun to point toward the developmental consequences of “emotional neglect” (Cicchetti & Toth, 2005), yet this term often remains underdefined or misinterpreted in professional practice. The construct becomes even more elusive in cases where children do not identify themselves as victims or do not meet institutional thresholds for intervention. Consequently, a significant segment of vulnerable children may be systematically overlooked, not because their suffering is absent but because it lacks a clear name.

While prior research has established the role of disrupted attachment and adverse childhood experiences in shaping later vulnerability (Bowlby, 1988), limited attention has been paid to the more covert and culturally normalized forms of parental emotional absence. These absences, which are less visible than overt abuse or material neglect, may be internalized by children as developmentally “normal,” thereby evading detection both by professionals and by the children themselves (Fonagy et al., 2002).

Furthermore, there is a lack of research capturing how these experiences are articulated by both survivors and frontline professionals in contexts where trafficking risk is high, such as Albania and the broader Balkan region. This study builds upon a growing body of qualitative research that explores child trafficking not solely as an event but as a process rooted in developmental vulnerabilities.

In particular, it draws from interviews with both survivors of trafficking and professionals in Albania, aiming to understand the early relational scripts that shape susceptibility to manipulation and control. Unlike previous studies that focus on the material or behavioral risk factors, this research foregrounds emotional experiences and the internalized belief systems they foster.

We examine three interrelated themes—emotional absence, parentification, and silencing—as core features of developmental disruption. Our aim is to demonstrate how these lived realities form a psychological infrastructure of vulnerability: one in which the self is not protected, not prioritized, and not allowed to express need. By investigating these emotional and relational patterns through the voices of those who lived and witnessed them, we seek to contribute to a more nuanced, trauma-informed understanding of trafficking vulnerability, one that begins not at the point of contact with exploiters but within the silences of the family home.

This study seeks to address this gap by asking:

How is parental emotional absence described by survivors and professionals, and in what ways might this subtle but persistent absence contribute to developmental vulnerability to trafficking?

## 2. Literature Review

The literature on child trafficking has increasingly acknowledged the role of relational and psychological vulnerability, in addition to structural factors such as poverty, institutional neglect, and gender-based violence (Cicchetti & Toth, 2005). While socio-economic conditions and systemic failures remain critical to understanding exposure to trafficking, emerging psychological frameworks suggest that early disruptions in caregiving relationships may underlie a developmental predisposition to exploitation (Courtois & Ford, 2009).

In particular, the experience of emotional neglect, often unarticulated by victims and underestimated by professionals, has received limited empirical attention in the context of trafficking vulnerability. Attachment theory provides one of the most influential lenses through which early caregiver–child dynamics have been conceptualized. Bowlby (Bowlby, 1969) emphasized that consistent emotional attunement forms the foundation for a child’s sense of safety, trust, and autonomy.

Disruptions in these bonds, whether through abandonment, rejection, or affective unavailability, may produce insecure or disorganized attachment patterns that endure into adulthood and increase susceptibility to harmful relationships. Several scholars have expanded this understanding by highlighting the long-term effects of “invisible wounds” such as emotional neglect, invalidation, or chronic misattunement (Gilligan, 1982).

However, emotional neglect often lacks the discursive or diagnostic clarity of more overt forms of abuse. It rarely leaves physical marks, nor does it always provoke an institutional response. As a result, children raised in such environments may internalize their experience as normative, learning to suppress emotional needs or derive self-worth from compliance, self-silencing, or premature caregiving roles.

Research in developmental psychopathology has linked these early dynamics to later difficulties in recognizing danger, setting boundaries, and seeking help—traits that may heighten vulnerability to grooming and coercion (Cicchetti & Toth, 2005). The concept of relational trauma (Herman, 1992) further deepens this analysis by centering the developmental consequences of being emotionally unseen or unheard during critical periods of growth.

Traumatologists argue that when affective needs are persistently denied or punished, children may learn to anticipate rejection, numb emotional states, and seek validation through relational self-abandonment. In contexts of poverty or parental mental health problems, these dynamics may become entrenched rather than episodic, shaping not only internal working models but also external relational scripts.

Recent contributions by Gabor Maté (Maté, 2022) and Van der Kolk (2014) have illuminated the neurobiological and affective implications of chronic emotional dysregulation and insecure attachment. Their work suggests that prolonged emotional absence or fragmentation during childhood impacts the development of self-regulatory capacities and stress responses—both essential for assessing interpersonal risk and maintaining autonomy in exploitative dynamics.

While their analyses are not trafficking-specific, they provide a theoretical foundation for understanding how such vulnerabilities may function as a “preconditioned landscape” for exploitation.

In parallel, feminist and socio-relational perspectives have emphasized how gendered expectations shape the normalization of silence, self-effacement, and emotional labor in girls (Gilligan, 2021). In patriarchal or transitional societies such as those in the Western Balkans, these expectations may further compound risk, as emotional needs are framed as weakness and care becomes a duty rather than a right.

This context may explain why many victims do not perceive themselves as “neglected” but as failing to adapt to familial or societal roles. The Albanian socio-cultural context further shapes the expression and perception of emotional neglect. High cultural value placed on resilience, coupled with stigma around emotional expression, particularly for girls, can normalize silence and self-effacement as virtues rather than warning signs.

In patriarchal family structures, caregiving expectations for female children often include managing household and emotional labor from an early age, blurring the boundaries between care and self-sacrifice. For boys, cultural scripts may discourage vulnerability and prioritize stoicism, limiting their capacity to articulate distress or seek support. These gendered patterns, embedded in post-transition social and economic instability, create fertile ground for the unnoticed perpetuation of relational harm.

Despite these contributions, there remains a significant gap in research that directly examines how emotional neglect, especially when culturally normalized, emerges in the life narratives of trafficking survivors and how professionals interpret such histories. The present study aims to contribute to this literature by exploring these under-articulated relational dimensions in a context where structural and emotional vulnerabilities often overlap, yet where institutional interventions remain largely reactive and symptom-based.

In addition to theoretical perspectives, international policy frameworks emphasize the importance of early identification and prevention of trafficking risk. The United Nations Palermo Protocol (United Nations, 2000) and the Council of Europe Convention on Action against Trafficking in Human Beings (Council of Europe, 2005) both highlight the need to address not only structural and economic drivers but also relational vulnerabilities within the family.

These instruments, alongside the Group of Experts on Action against Trafficking in Human Beings (GRETA, 2022) evaluations, underscore the integration of psychosocial assessment in child protection systems. In Albania, the National Anti-Trafficking Strategy (2021–2023; Ministry of Interior, 2021) recognizes family breakdown and deficient caregiving as contributing factors to child exploitation, yet implementation has remained predominantly reactive and incident-driven rather than preventive.

In terms of applied implications, the literature increasingly points to the value of trafficking-specific intervention models. Trauma-informed community policing, early childhood developmental screening, and family-based attachment interventions have demonstrated potential in identifying relational risk factors before exploitation occurs (Treisman, 2021).

Integrating such models into national child protection protocols, in alignment with Albania’s existing legal and policy commitments, could bridge the gap between recognition of relational harm and timely preventive action.

## 3. Materials and Methods

This study adopted a qualitative, interpretive research design informed by a developmental–relational epistemology. Vulnerability was conceptualized not as a static or purely structural risk factor but as a relational trajectory shaped by early caregiving dynamics, emotional silencing, and the internalization of fractured attachment patterns. This epistemological stance privileges the subjective and affective dimensions of experience, recognizing that meaning-making emerges within relational and developmental contexts.

While this approach enables a nuanced exploration of how vulnerabilities accumulate across time, it also acknowledges the interpretive subjectivities inherent in qualitative inquiry. Reflexive memoing and regular peer discussions were employed throughout the analysis to mitigate personal biases and ensure that emergent themes reflected participants’ lived experiences rather than researcher projections.

### 3.1. Participants and Sampling Strategy

Data collection involved a total of 30 primary participants, representing two complementary perspectives on vulnerability factors.

- Professionals: Sixteen individuals (psychologists, social workers, and legal staff) participated through two focus groups (*n* = 9 and *n* = 7) and additional individual semi-structured interviews.
- Survivors and victims of trafficking: Fourteen adults were identified by service providers as either survivors of sexual trafficking or individuals currently trafficked. They were interviewed using a trauma-informed protocol implemented by trained professionals within partner services. Participants included women (*n* = 9) and transgender women (*n* = 5), aged 18–36.

Survivor participants were identified through professional referrals within six established service organizations across Albania, all of which maintain ethical review protocols for engaging clients in research. Eligibility criteria included clinical stability, informed consent capacity, and emotional readiness, assessed jointly by service providers and research team members trained in trauma-informed interviewing.

No incentives were offered in order to ensure participation was voluntary. Participants were not contacted directly by researchers; rather, they were approached by their caseworkers, who explained the study and conveyed interest back to the team only upon explicit participant consent.

A purposive sampling strategy was employed to access both narrative-rich professional insights and direct accounts of lived experience. The inclusion of transgender women, often overlooked in trafficking discourse, added critical perspectives on gendered vulnerability.

Survivors and currently trafficked participants were treated on equal analytical footing. Survivors’ narratives offered retrospective insights into long-term developmental trajectories, while those from currently trafficked individuals reflected ongoing relational dynamics. Both groups contributed to the same thematic framework, with attention paid to temporal framing and immediacy of experience.

Data saturation was reached when interviews consistently reiterated core relational themes of emotional silencing, conditional recognition, and fractured attachment without yielding substantially new dynamics. Saturation was understood not as an absolute endpoint but as an indicator of thematic stability across both datasets.

### 3.2. Data Collection Procedures

Data were collected between December 2024 and March 2025 in collaboration with six service organizations across Albania. A semi-structured protocol guided all interviews and focus groups, ensuring thematic consistency while allowing flexibility for emergent narratives.

All survivor interviews were conducted only with individuals deemed clinically stable and emotionally safe to participate. Informed consent procedures were designed to preserve agency and minimize coercion. Interviews with currently trafficked persons were conducted in secure environments, ensuring maximum feasible safety and confidentiality, with the option to withdraw at any point without consequence.

Focus groups lasted approximately 90 min, and individual interviews ranged from 30 to 60 min. All sessions were audio-recorded with participant permission and transcribed verbatim for analysis. Reflexive field notes were maintained to capture non-verbal cues and contextual factors influencing the interviews.

Interviewers had prior clinical training in trauma-focused therapy and certification in ethical interviewing practices for vulnerable populations. They attended preparatory workshops on managing retraumatization risk, dissociation cues, and emotional regulation techniques. Psychological first aid was available on-site, and post-interview emotional check-ins were conducted.

Ongoing debriefings between researchers and clinical supervisors ensured sustained ethical vigilance throughout data collection.

### 3.3. Data Analysis

Data were analyzed using reflexive thematic analysis (Braun & Clarke, 2006), privileging both semantic content and latent emotional meanings embedded within participant narratives. Interviews were treated as holistic texts, allowing a comprehensive examination of overlapping emotional, relational, and symbolic patterns.

Two researchers independently coded transcripts, followed by iterative discussions to reconcile interpretive tensions. Reflexive memos documented the researchers’ positionalities and emotional reactions, increasing transparency and critical engagement with the data.

Themes were conceptualized not as static categories but as interrelated developmental–relational processes operating below institutional recognition thresholds. Special attention was given to patterns showing how emotional silencing, attachment fractures, and systemic failures cumulatively shaped vulnerability trajectories.

## 4. Findings: Emotional Absence as a Normalized Terrain

Thematic analysis revealed a pervasive pattern across survivor and professional narratives: parental emotional absence was not described as an exceptional trauma but as a normalized condition shaping expectations of care, self-worth, and relational safety. This absence appeared in families that provided food, order, and shelter but little recognition of emotion. Children adapted by silencing their own needs and, in some cases, by caring for the emotions of adults.

Although commonalities were strong, experiences varied. Many participants described consistent emotional neglect across childhood; some recalled temporary warmth followed by renewed silence, and a few connected emotional withdrawal to migration, loss, or parental mental health. This variability shows that emotional absence existed on a continuum rather than as a single experience.

### 4.1. “They Were There, but I Wasn’t Seen”

Several survivors described their childhood homes as emotionally sterile, though materially secure:

*“My mother made food, kept the house clean, and told me to behave. But I don’t remember once being asked how I felt. I think I stopped crying around age seven.”*

*“At home we didn’t talk about feelings. If something hurt, you were told to be strong. Crying was shameful.”*

*“My father was kind but distant. I remember him reading the newspaper while I tried to show him my drawings. He nodded but didn’t look.”*

Professionals working with youth in community centers also noted this pattern:

*“We often meet adolescents who have never been abused, yet they feel invisible at home. They describe parents who provide but do not notice.”*

(Social worker, Tirana)

*“The absence of curiosity toward children’s feelings is so normalized that many parents see emotional talk as weakness.”*

(Child psychologist, Elbasan)

Professionals confirmed that such experiences were rarely recognized as neglect:

*“They often don’t know they were neglected; they think needing attention is wrong or weak.”*

(Social worker, Tirana)

*“Many adults who were trafficked describe childhoods without visible abuse just no one noticing them.”*

(Psychologist, Shkodër)

Across narratives, care equaled duty rather than presence. What survivors remembered was not violence but indifference, a quiet message that emotions were unnecessary. Over time, this emotional stillness became the expected mode of relating to adults. What began as coping gradually evolved into a life pattern of restraint and self-control.

### 4.2. Early Parentification and Emotional Inversion

A second theme was emotional inversion, in which children assumed adult roles to maintain family balance. Survivors often described becoming emotional caregivers for parents struggling with trauma or instability:

*“I learned early to read her moods, to avoid upsetting her. If she was okay, then I could be okay.”*

*“My mother cried a lot, so I would make her coffee and tell her not to worry. I was ten.”*

*“In our house, I was the one who comforted others. No one ever asked if I needed something.”*

Several professionals recognized this silence during therapy sessions:

*“You can sense the quietness in their posture before they speak. They apologize for having feelings.”*

(Trauma therapist, Durrës)

Professionals observed that such parentified children later showed hyper-responsibility and difficulty setting boundaries:

*“They come to therapy saying ‘I can’t rest if someone around me is upset.’ They grew up believing love means fixing others.”*

(Counselor, Durrës)

This theme appeared across genders, though it was more pronounced among girls and transgender women, who linked care with worthiness and silence.

### 4.3. Silencing as Survival

Silence, both spoken and embodied, emerged as a survival strategy. Participants recalled that expressing distress invited punishment or ridicule:

*“If I said something, it was drama. So I stopped talking. I still find it hard to say what I need.”*

*“When I cried, they laughed. They said, ‘You’re too sensitive.’ I learned to smile even when I wanted to scream.”*

*“In our house, peace meant no one talking. We walked quietly, like guests in our own home.”*

Professionals confirmed this pattern of learned quietness:

*“Many survivors describe the body as silent tense shoulders, shallow breathing. It’s a way of staying safe.”*

(Psychotherapist, Tirana)

Over time, silence transformed from protection into identity. Participants described it as a habit that followed them into adulthood, useful for survival but costly to authenticity.

An overview of the identified themes is presented in Table 1.

The voices of survivors and professionals converge around a quiet, pervasive harm—emotional absence as a daily routine rather than a dramatic event. The following discussion interprets these narratives within a developmental and cultural framework, examining how emotional absence, parentification, and silencing interact to shape long-term vulnerability.

## 5. Discussion

The findings of this study reveal that emotional absence, parentification, and silencing are interwoven dynamics that structure developmental vulnerability. What survivors describe is not overt abuse but the normalization of emotional invisibility, an experience of being “present but unseen.” These three themes form a self-reinforcing cycle: absence fosters role reversal, maintained through silence, which in turn conceals the absence itself.

This discussion interprets these narratives through developmental and cultural frameworks, situating them within the broader literature on trauma, attachment, and relational harm.

### 5.1. Interpreting Emotional Absence

The recurrent description of children who “stopped crying around age seven” captures a crucial developmental moment: the internalization of futility. The child learns that emotional expression has no relational outcome. This aligns with Gilligan’s (1982) notion of relational disconnection, where silence preserves attachment in the face of unresponsiveness.

Professionals’ observations like “They think needing attention is wrong or weak” illustrate the same process from a clinical perspective: a self-blame mechanism that replaces the expectation of care.

From a neurodevelopmental lens, Schore (2001) and Fonagy et al. (2002) argue that consistent emotional attunement co-regulates affect and builds the neural architecture for trust. Its absence results in hypervigilance and relational uncertainty. In the Albanian socio-cultural context, where stoicism is a moral ideal, emotional absence is not perceived as neglect but as strength. This helps explain why many participants could not name it as harm.

### 5.2. Interpreting Parentification and Emotional Inversion

The repetition of “okay” in survivor accounts (“If she was okay, then I could be okay”) encapsulates emotional inversion: the child’s safety becomes dependent on the caregiver’s stability. Maté (2003, 2022) describes this as a “compulsion to self-abandon,” a learned belief that love and approval require suppressing personal need.

This finding mirrors global evidence that parentification correlates with later caregiving in adult relationships (Chase, 1999), yet here it acquires a distinctly cultural tone. Within Albania’s post-transition context, where endurance and sacrifice are valorized, this inversion is socially rewarded. Being “a good child” often meant emotional silence and compliance qualities later exploited in grooming dynamics.

Gender norms magnify this process. Gilligan (2021) notes that women’s relational identities are often moralized through care and self-denial. In survivor narratives, girls and transgender women described responsibility for others’ emotions as a feminine duty. Thus, cultural scripts of gender merge with psychological vulnerability to create cumulative developmental risk.

### 5.3. Silencing and the Physiology of Survival

Silence was described as both protection and identity. This duality aligns with Herman’s (1992) concept of chronic captivity and Porges’ (2011) polyvagal theory, which link emotional suppression to physiological hyperarousal and dissociation.

In participants’ narratives, such as “I still find it hard to say what I need” and “Peace meant no one talking,” silence is embodied. Perry (2021) observes that such chronic shutdown recalibrates the nervous system to perceive connection as unsafe. For many survivors, this bodily imprint made coercive relationships feel familiar rather than dangerous.

Professionals corroborated this, describing the “quiet body” in therapy as a posture of self-containment, signaling a lifetime of adapting to emotional neglect. Thus, silence, while initially protective, evolves into a relational template that perpetuates vulnerability.

### 5.4. Cultural Context and Societal Normalization

A key contribution of this study lies in contextualizing emotional neglect within Balkan cultural scripts. Emotional absence is reinforced by collective values that equate endurance with dignity and dependence with weakness. Within this logic, parents see emotional control as a virtue, not a failure.

This normalization extends to institutions: schools, workplaces, and even mental health systems rarely name emotional deprivation as a problem. Maté (2022) argues that modern societies reward disconnection disguised as functionality. In Albania’s transitional landscape, this disconnection is inherited a quiet residue of intergenerational trauma and economic uncertainty.

Understanding vulnerability to trafficking, therefore, requires a cultural as well as psychological lens. The relational dynamics described here are not individual pathologies but socially sanctioned forms of emotional austerity.

### 5.5. Implications for Trauma-Informed Practice

The results call for an expansion of trauma-informed frameworks beyond visible violence to include normalized emotional neglect. Preventive work should evaluate relational safety, the degree to which a child’s emotions are recognized and validated, as seriously as material welfare.

Practical steps include:

- Attachment-based family assessments integrated in child protection protocols;
- Early screening in preschools for signs of emotional withdrawal or role reversal;
- Training for professionals to detect culturally normalized neglect and gendered expectations;
- Trauma-informed community policing to identify relational risk before exploitation occurs.

By addressing emotional safety as a core developmental right, interventions can shift prevention from reactive protection to proactive relational care.

### 5.6. Limitations and Power Dynamics

Although interviewers had no prior therapeutic relationship with participants, their professional status may have influenced disclosure. This relational asymmetry, coupled with the sensitive topic, might have shaped what was said and what remained unspoken.

The study’s aim is depth and transferability, not generalizability. Its insights are bound to the Albanian context but may resonate with other societies where silence is cultural capital. Future work should explore participatory and longitudinal designs to trace how early emotional patterns evolve into adult relational risk.

### 5.7. Integrative Reflection

Together, the three themes illuminate a developmental paradox: children adapt to emotional scarcity by becoming self-sufficient, yet this very adaptation erodes their ability to seek help later. Emotional absence, parentification, and silencing are thus not discrete categories but a continuum of relational harm.

Recognizing this continuum reframes prevention not as rescuing from external threats, but as rebuilding internal safety where the capacity for emotional connection was once lost.

## 6. Conclusions

Chronic, normalized emotional absence within families quietly structures children’s susceptibility to trafficking. Such harm often goes unnamed, leaving children to adapt through silence, compliance, and self-effacement patterns later exploited because they mirror familiar family dynamics.

Prevention and intervention should therefore:

- Integrate family-based attachment assessments within child protection protocols;
- Train service providers to recognize culturally normalized neglect;
- Implement developmental and attachment screening in preschool settings;
- Adopt trauma-informed policing models to identify relational risk early;
- Embed cultural competence in prevention, aligning with Albanian socio-cultural realities.

Limitations: This study’s sample size (*N* = 30) limits generalization but provides depth and transferability. As a qualitative study, findings reflect lived experiences rather than statistical representativeness. Context specificity and retrospective accounts introduce potential bias, yet the results offer valuable insight into relational harm as a developmental risk factor.

## Figures and Tables

**Table 1:** Overview of themes and subthemes emerging from the data.

Main Themes	Subthemes	Illustrative Meaning
Emotional absence	Parental disengagement; muted attunement	Presence without emotional recognition
Parentification	Emotional inversion; adultified responsibility	Child regulates parents’ affect; loss of developmental space
Silencing	Learned voicelessness; shame of expression	Emotion equated with conflict; survival through quietness

## Data Availability

The data supporting the findings of this study are not publicly available due to confidentiality and ethical restrictions. Anonymized excerpts of the qualitative material are available from the corresponding authors upon reasonable request.
